# The Enemata of Wine

**Published:** 1876-08

**Authors:** 


The Enemata of Wine.—In the *Revue Medicale* M. Baraillier calls attention to the great utility of vinous enemata in all slow and difficult convalescences, colliquative diarrhœa, obstinate leucorrhœa, chlorosis, and dyspepsia with vomiting. One or two enemata, of from 150 to 200 grammes (℥v to ℥viij) of the red wine of Provence are given once or twice a day, at a tepid temperature; and, the intestine being first emptied by a tepid enema, the action of the wine is speedily manifested, especially in persons not given to alcoholic drinks, and therefore in women and children. If, as it does sometimes, it prove too irritating, a third or half of water or syrup should be added. *Medical and Surgical Reporter.*

